# Adipokines and Myokines as Markers of Malnutrition and Sarcopenia in Patients Receiving Kidney Replacement Therapy: An Observational, Cross-Sectional Study

**DOI:** 10.3390/nu16152480

**Published:** 2024-07-31

**Authors:** Sylwia Czaja-Stolc, Antoine Chatrenet, Marta Potrykus, Jakub Ruszkowski, Massimo Torreggiani, Monika Lichodziejewska-Niemierko, Alicja Dębska-Ślizień, Giorgina Barbara Piccoli, Sylwia Małgorzewicz

**Affiliations:** 1 Department of Clinical Nutrition and Dietetics, Faculty of Health Sciences, Medical University of Gdansk, 80-211 Gdansk, Poland; sylwia.czaja-stolc@gumed.edu.pl (S.C.-S.); sylwia.malgorzewicz@gumed.edu.pl (S.M.); 2 Department of Nephrology, Centre Hospitalier du Mans, 72037 Le Mans, France; antoine.chatrenet@gmail.com (A.C.); maxtorreggiani@hotmail.com (M.T.); gbpiccoli@yahoo.it (G.B.P.); 3 APCoSS—Institute of Physical Education and Sports Sciences (IFEPSA), UCO, 49136 Angers, France; 4 Department of Oncological, Transplant, and General Surgery, Faculty of Medicine, Medical University of Gdansk, 80-214 Gdansk, Poland; 5 Department of Nephrology, Transplantology and Internal Medicine, Faculty of Medicine, Medical University of Gdansk, 80-214 Gdansk, Poland; jakub.ruszkowski@gumed.edu.pl (J.R.); alicja.debska-slizien@gumed.edu.pl (A.D.-Ś.); 6 Department of Palliative Medicine, Faculty of Medicine, Medical University of Gdansk, 80-211 Gdansk, Poland; monika.lichodziejewska-niemierko@gumed.edu.pl; 7 Department of Nephrology, University of Angers, 49035 Angers, France

**Keywords:** chronic kidney disease, adipokines, myokines, sarcopenia, malnutrition, myostatin

## Abstract

Chronic kidney disease (CKD) is linked to an elevated risk of malnutrition and sarcopenia, contributing to the intricate network of CKD-related metabolic disorders. Adipokines and myokines are markers and effectors of sarcopenia and nutritional status. The aim of this study was to assess whether the adipokine–myokine signature in patients on kidney replacement therapy could help identify malnutrition and sarcopenia. The study involved three groups: 84 hemodialysis (HD) patients, 44 peritoneal dialysis (PD) patients, and 52 kidney transplant recipients (KTR). Mean age was 56.1 ± 16.3 years. Malnutrition was defined using the 7-Point Subjective Global Assessment (SGA) and the Malnutrition-Inflammation Score (MIS). Sarcopenia was diagnosed based on reduced handgrip strength (HGS) and diminished muscle mass. Concentrations of adipokines and myokines were determined using the enzyme-linked immunosorbent assay (ELISA). 32.8% of all study participants were identified as malnourished and 20.6% had sarcopenia. For malnutrition, assessed using the 7-Point SGA, in ROC analysis albumin (area under the curve (AUC) 0.67 was the best single biomarker identified. In dialysis patients, myostatin (AUC 0.79) and IL-6 (AUC 0.67) had a high discrimination value for sarcopenia, and we were able to develop a prediction model for sarcopenia, including age, albumin, adiponectin, and myostatin levels, with an AUC of 0.806 (95% CI: 0.721–0.891). Adipokines and myokines appear to be useful laboratory markers for assessing malnutrition and sarcopenia. The formula we propose could contribute to a better understanding of sarcopenia and potentially lead to more effective interventions and management strategies for dialysis patients.

## 1. Introduction

Chronic kidney disease (CKD) is a global health issue currently affecting approximately 12% of individuals worldwide [1]. The number of patients requiring kidney replacement therapy, namely dialysis and kidney transplantation (KT), is increasing [2].

Malnutrition, defined as insufficient nutrient intake and absorption, which results in altered body composition, is highly prevalent in CKD, affecting over 60% of patients [3,4]. The cumulative reduction in muscle mass and muscle force, called sarcopenia, is also widespread in the CKD population, ranging from 15 to 35% [5]. These disorders are closely associated with an increased risk of death [6]. In patients on dialysis, decreased appetite is frequent and is associated with uremic intoxication, metabolic acidosis, and inflammation [7,8,9]. In addition, strict dietary restrictions may further limit food intake. Furthermore, fatigue is frequent in patients with end-stage kidney disease, and the consequent reduction in physical activity increases muscle loss and sarcopenia. During dialysis sessions, there is a loss of amino acids, which may intensify the catabolic effect of uremia and dialysis itself [10,11]. Conversely, after KT, appetite usually improves and dietary restrictions are loosened, which results in increased food consumption, and sometimes in an impressive weight gain. However, since steroids and calcineurin inhibitors increase the risk of diabetes, different dietary restrictions are usually advised [12].

In patients with advanced CKD, the metabolic balance in insulin-dependent tissues increases muscle catabolism, leading to sarcopenia, and at the same time increasing anabolism in fat tissue. While the clinical elements characterizing progressive muscle loss in CKD are well identified, the molecular pathways are only partially known. Signaling factors secreted by muscle tissue (i.e., myokines) and by adipose tissue (i.e., adipokines) appear to influence nutritional status [13,14]. The complex network connecting adipokines, myokines, and the nutritional status of patients with CKD is not fully understood.

In CKD, elevated levels of adipokines, notably leptin and adiponectin, contribute to altered appetite regulation and systemic inflammation. While leptin diminishes appetite via hypothalamic signals, adiponectin’s anti-inflammatory and metabolic properties are compromised in CKD, leading to protein-energy wasting (PEW) and increased mortality risk [15,16]. Skeletal muscle wasting, prevalent in CKD due to metabolic acidosis, insulin resistance, and inflammation, involves myostatin contributing to muscle protein synthesis inhibition and degradation [17,18]. Lower levels of irisin in CKD patients are linked to cardiovascular mortality, possibly due to reduced muscle volume [19,20]. The main characteristics of adipokines and myokines, and available data for CKD patients, are summarized in Table 1.

There are indications that the balance between adipokines and myokines is altered in patients with advanced CKD and those on kidney replacement therapy (KRT) and that this imbalance may modulate their nutritional status and represents a nutritional metabolic signature. In all recent major guidelines, nutritional status evaluation plays a pivotal role [21,22]. There exist a number of biochemical and instrumental tests to assess muscular mass as a determinant of nutritional status. However, the performance and interpretation of these evaluations depend on local expertise and rely on a multidisciplinary team evaluation. Performing a nutritional assessment requires time, experienced staff, and equipment. Laboratory tests are likely to be more accessible than dietary consultations. Thus, in settings where a dietician is not available or nephrologists do not have longstanding expertise in nutritional assessment, a further objective determination of nutritional status by dosing biochemical markers other than those commonly suggested by the guidelines may help to select patients needing a more extensive evaluation. For this reason, we undertook this study to verify whether or not the adipokine and myokine signatures can be used as reliable markers of malnutrition and sarcopenia.

**Table 1:** Some reference data on adipokines and myokines.

	Reference Values in Healthy Individuals	Reports on CKD Patients with Good Nutritional Status	Reports on CKD Patients with Poor Nutritional Status	Reports on Sarcopenia in CKD Patients	Key Functions
Leptin	0.33–19.85 ng/mL in men and 3.60–54.86 ng/mL in women [23]	↑	↓	↓	Regulates appetite, signals satiety, decreases food intake [16,24]
Adiponectin	2–20 μg/mL [25]	↑	↑	Data lacking	Plays a role in energy homeostasis, anti-inflammatory effects [15,24]
IL-6	<5.740 pg/mL [26]	↑	↑	↑	Pro-inflammatory effect; contributes to muscle protein breakdown and can impact appetite regulation [24,27]
Myostatin	7–32 ng/mL [28]	↑	↑↓ non-conclusive results	↑↓ non-conclusive results	Muscle protein synthesis inhibition; contributes to muscle atrophy [29,30]
Irisin	5.1–62.7 μg/mL [31]	↓	↑↓ non-conclusive results	↑↓ non-conclusive results	Associated with thermogenesis, involved in muscle protein synthesis [18,32]

Legend: ↑, higher concentration; ↑, lower concentration; ↑↓, contradictory results

## 2. Materials and Methods

### 2.1. Study Design

This observational, cross-sectional study aimed to evaluate the profiles of adipokines and myokines and to assess their role as biomarkers of malnutrition and sarcopenia. To test this hypothesis, 3 groups of patients on kidney replacement therapy were studied: hemodialysis (HD) patients, peritoneal dialysis (PD) patients, and kidney transplant recipients (KTR). A group of healthy individuals was recruited to validate normal test results.

The diagnostic yield of different adipokines and myokines was tested against a multidimensional standardized nutritional evaluation that included anthropometric tests, body composition analysis, the 7-Point Subjective Global Assessment (SGA), and the Malnutrition-Inflammation Score (MIS).

The study was approved by the Independent Bioethical Committee of the Medical University of Gdansk (NKBBN/613/2020, 24 November 2020).

### 2.2. Study Population

Enrolment took place at the University Clinical Center in Gdansk (Poland) from March to December 2021. All HD and PD patients receiving treatment were told about the study by the center’s dietician. Outpatients with KT were told about the study by one of the physicians at the transplant clinic.

**Figure 1:**
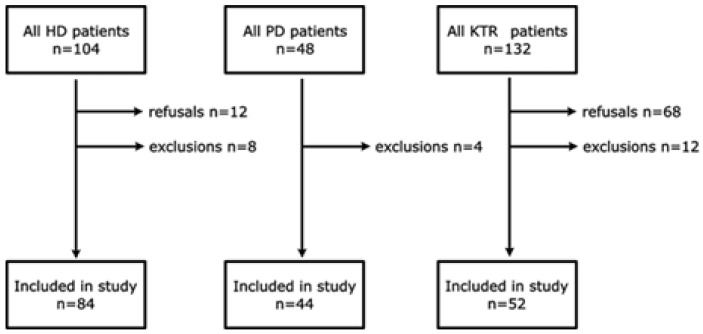
Flow chart of the study population. Legend: HD, hemodialysis; PD, peritoneal dialysis; KTR, kidney transplant recipients.

The inclusion criteria for HD and PD patients were as follows: over 18 years of age, minimum 3 months dialysis vintage, and written informed consent to participate. The inclusion criteria for KTR were as follows: over 18 years of age and a minimum of 3 months since transplantation, stable clinical condition (no recent surgical or infectious complications linked to KT, including signs of allograft rejection). The exclusion criteria were inability to give informed consent, cognitive impairment, and active oncological diseases. Recruiting KTR was difficult, as it involved scheduling additional hospital visits during the COVID-19 pandemic. The flow chart of the study is presented in Figure 1.

#### 2.2.1. Standard Treatment

HD patients were treated with high-flux membranes (Helixone), with surface areas between 1.4 and 2.2 m^2^. All patients were on thrice-weekly HD, typically lasting 4 h, (range 3–4.5 h). Blood flow was maintained between 300 and 350 mL/min. Dialysis adequacy (Kt/V) was calculated using the second-generation Daugirdas formula: Kt/V = −Ln((BUN postdialysis/BUN pre-dialysis) − (0.008 × hours)) + ((4 − (3.5 × BUN postdialysis/BUN pre-dialysis)) × UF volume/Weight postdialysis (LN, natural logarithm, BUN, blood urea nitrogen, UF, ultrafiltration) [33].

On PD, for patients undergoing continuous ambulatory peritoneal dialysis (CAPD), the standard treatment involved 4 daily exchanges using a 2000–2500 mL solution with low glucose content, while individuals on automated peritoneal dialysis (APD) received 10,000 mL of a low-glucose solution throughout the night. The volume of fluid completely cleared of urea in a single dialysis session (Kt) was assessed using the formula Kt [L] = (dialysate urea [mg/dL]/plasma urea [mg/dL]) × drain volume [L] [34]. The volume of distribution of urea (V) was calculated using the Watson Equation [35]. Kt/V is expressed as weekly values (total daily values multiplied by 7) [34].

For KTRs, the standard triple immunosuppressive treatment included glucocorticosteroids, a calcineurin inhibitor, and mycophenolate mofetil.

#### 2.2.2. Validation of Reference Values—Control Group

A control group was included to validate in our setting the reference values reported in the literature. The healthy volunteers were invited to participate through an announcement posted on social media or were contacted by direct university or hospital staff. The inclusion criteria were being over 18 years of age, absence of chronic diseases or chronic medications, and an estimated glomerular filtration rate (eGFR) calculated using the Chronic Kidney Disease Epidemiology Collaboration equation (CKD-EPI), >60 mL/min/1.73 m^2^ [36]. The control group consisted of 30 healthy volunteers, 15 women and 15 men, with an average age of 49 ± 9.5 years.

### 2.3. Data Collection

Baseline data, including sex and age, dialysis vintage, time elapsed since start of KT, and specific causes of kidney failure, were retrieved from patients’ medical charts.

### 2.4. Anthropometric Measurements

For HD patients, body weight was assessed pre- and post-dialysis. The post-dialysis weight was used for analysis. PD patients had their weight measured following a drain. For the remaining study participants, body weight was recorded in the morning, after overnight fasting. Body Mass Index (BMI) was determined by dividing weight in kilograms by squared height in meters. Waist and hip circumference were measured. Mid-arm circumference (MAC) and calf circumference (CC) measurements were performed on non-dominant limbs. According to the Mini Nutritional Assessment (MNA), MAC < 22 cm and CC < 31 cm were considered indicative of reduced muscle mass [37].

Handgrip strength (HGS) in the non-dominant hand was assessed using a dynamometer (Baseline^®^ Smedley, Fabrication Enterprises, New York, NY, USA). For HD patients with an arterio-venous fistula, HGS was measured on the contralateral limb. Each participant squeezed the dynamometer with maximum effort twice. The average result was taken into account for the analysis. Norms for HGS according to gender complied with the indications of the European Working Group on Sarcopenia in Older People 2 (EWGSOP2) [38].

### 2.5. Body Composition

Body composition analysis was carried out with electrical bioimpedance, using the Body Composition Monitor manufactured by Fresenius Medical Care (Bad Homburg, Germany), which measures the frequency range from 5 to 1000 kHz. In HD patients, the measure was performed 15 min after dialysis, and in PD patients after a drain. In the remaining study participants, body composition analysis was performed in the morning, after overnight fasting. The examination was performed in the supine position. The measurement lasted 2 min.

The following measures were retrieved and analyzed: over-hydration (OH), total body water (TBW), intracellular water (ICW), extracellular water (ECW), lean tissue mass (LTM), fat mass (FAT), adipose tissue mass (ATM), and body cell mass (BCM).

All assessments were performed by the same trained dietician cooperating with the Department of Nephrology at the University Clinical Center in Gdansk.

### 2.6. Assessment of Malnutrition and Malnutrition-Inflammation Complex Syndrome

Malnutrition [3] was diagnosed in patients who scored ≤5 points in the 7-Point SGA [39]. Malnutrition-Inflammation Complex Syndrome (MICS) was assessed using the MIS. MICS was defined as an MIS score of 8 or higher [40].

For BMI, we considered a value under 23 kg/m^2^ as “low”, in accordance with the guidelines of the International Society of Renal Nutrition and Metabolism (ISRNM) [41]. Obesity was diagnosed based on the World Health Organization (WHO) classification as BMI ≥ 30 kg/m^2^ [42]. A decreased albumin value was defined as <3.8 g/dL in accordance with the ISRNM guidelines [41].

Sarcopenia was defined as decreased strength and muscle mass [5]. Reduced muscle strength was defined as a hand grip strength (HGS) of <16 kg for women and <27 kg for men [38]. Lean tissue index (LTI) < 14 kg/m^2^ was considered reduced [43] since the body composition analysis device used does not measure appendicular skeletal muscle mass (ASM). FTI was considered increased/decreased according to the algorithm proposed by the Body Composition Monitor used, adjusted for age and gender.

Sarcopenic obesity was diagnosed based on the consensus of the European Society for Clinical Nutrition and Metabolism (ESPEN) and the European Association for the Study of Obesity (EASO) [44]. The diagnostic criteria included the co-occurrence of obesity (BMI ≥ 30 kg/m^2^), low muscle mass (LTI < 14 kg/m^2^), and reduced muscle strength (HGS < 27 kg for males and <16 kg for females).

### 2.7. Assessment of Biochemical Data

HD patients’ blood was collected after overnight fasting in the case of patients with a morning dialysis session or after a 4 h fast in the case of patients on dialysis in the afternoon or evening, and before and after a mid-week dialysis session. For the remaining participants, blood was collected in the morning following an overnight fast. Cell blood count, glucose, creatinine, blood urea nitrogen (BUN), urea, calcium, phosphorus, iron, high-sensitivity C-reactive protein (hsCRP), adipokines, and myokines were tested. Sodium, potassium, lipid profile, albumin levels, ferritin, transferrin, and parathyroid hormone (PTH) were also assessed.

For the analysis of leptin, adiponectin, irisin, myostatin, hsCRP, and IL-6, serum samples were stored at −80 °C and subsequently tested using the enzyme-linked immunosorbent assay (ELISA). Leptin levels were measured using the Leptin Sandwich ELISA Kit (DRG, EIA 2395). Adiponectin was assessed with the ELISA Human Total Adiponectin/Acrp30 assay (Quantikine, DRP300, Bio-Techne, Minneapolis, MN, USA). Myostatin was evaluated using the GDF-8/Myostatin ELISA Kit (Quantikine, DGDF80). Irisin levels were determined using the Irisin ELISA Kit (Immuniq, RAG018R, Żory, Poland), and IL-6 levels were quantified using the Human IL-6 ELISA Kit (Quantikine, D6050). Laboratory determinations were conducted following the manufacturers’ recommendations.

### 2.8. Statistical Analysis

For the statistical analysis, we employed Microsoft Office Excel 365, Statistica 13.3 (StatSoft, Cracow, Poland), GraphPad Prism 8.4.3, and RStudio 2023.06.0 (R 4.2.3 and the following packages: pROC (version 1.18.5) [45], dplyr 1.1.3 [46], readxl (version 1.4.3), and jamovi 2.4.8 (meddecide 0.0.2.26 module)).

If the measured variables followed a normal distribution, they are shown as the mean ± standard deviation (SD); otherwise, they are expressed as the median and first quartile (Q1)–third quartile (Q3). To determine normal distribution, evaluations of histograms and the Shapiro–Wilk test were used. The differences between groups were calculated using either ANOVA or the Kruskal–Wallis test and post-hoc analysis by the Holm method. The differences between each two groups were calculated using the Student *t*-tests or the Mann–Whitney U test, depending on their distribution. Categorical variables were compared using the chi-squared test or Fisher’s exact test. The correlation analysis was performed using the Spearman or Pearson method, as appropriate.

#### 2.8.1. Receiver Operating Characteristics—Biomarkers of Sarcopenia and Malnutrition

Receiver operating characteristic (ROC) curves were generated for each biomarker (adiponectin, albumin, IL-6, irisin, leptin, and myostatin) to test their performance in identifying malnutrition (defined according to the 7-Point SGA), MICS (defined according to MIS), and sarcopenia (defined as HGS < 16 kg for women or <27 kg for men and LTI <14 kg/m^2^). Area under the curve (AUC) and 95% confidence intervals (95% CI) were estimated using the variance of the AUC computed, as defined by DeLong et al. (1988) [47] using the algorithm by Sun and Xu (2014) [48]. If the lower bound of the 95% CI of AUC was higher than 0.5, the biomarker was considered significant. For each significant biomarker, the optimal threshold was established based on Youden’s J statistic, which maximizes both sensitivity and specificity. DeLong’s test (or bootstrap with 2000 replicates, if DeLong’s test was not applicable) was conducted to compare AUCs between groups (HD versus PD patients; dialysis-dependent patients versus KTRs) and within groups (the different biomarkers). If the AUC differed significantly between HD and PD groups, we did not combine dialysis-dependent patients. Moreover, if needed, to assess whether the two ROC curves were perfectly superimposed they were tested using Venkatraman’s model [49]. Sensitivity, specificity negative, and positive predictive power were calculated.

#### 2.8.2. Models Predicting Malnutrition and Sarcopenia

We tested to determine whether a combination of biomarkers yielded a better AUC than the individual components.

To build a model capable of predicting malnutrition and sarcopenia, the following variables were tested: age, sex, albumin, leptin, adiponectin, irisin, myostatin, and IL-6. The model was built using an augmented regression with backward elimination (significance threshold, α = 0.1, change-in-estimate threshold, τ = 0.05) [50] using the abe R package version 3.0.1., with a more conservative and stable final model built by reducing the false exclusion bias of confounding variables compared to the traditional backward regression model [50].

While in the case of malnutrition-MICS, no combination performed better than its individual biomarkers; this approach allowed us to build a model that performed better than any single biomarker in the identification of sarcopenia. The final model, including the retained variables, was used to build a formula, which was further tested through ROC curve analysis.

## 3. Results

### 3.1. Baseline Data and Nutritional Status

The baseline characteristics of the 180 study participants are presented in Table 2; we recruited 84 patients in the HD group, 44 in the PD group, and 52 in the KTR group. Among KTR, 43 patients had previously been on dialysis. The median dialysis vintage was 18 (10–36) months. The time since KT was 115.5 (53.5–192) months. The leading causes of CKD were glomerulonephritis and diabetic kidney disease (DKD).

Overall, 25.6% of participants exhibited reduced HGS values, more commonly in HD patients (41.7%). Among PD patients, 20.5% had reduced HGS, while in the KTR group the percentage was lower (3.8%).

LTI was decreased (i.e., <14 kg/m^2^) in 75.6% of participants and FTI was elevated in 21.1%. The highest prevalence of decreased LTI was observed in the KTR group, with 86.5%, followed closely by HD patients with 75%. Table 3 shows the results of the main laboratory tests.

### 3.2. Adipokines and Myokines

The concentrations of adipokines and myokines among healthy subjects were similar to the results of other studies conducted among healthy people: leptin concentration was 5.3 (2.4–16.2) ng/mL, adiponectin 2.6 (1.5–4.1) μg/mL, IL-6 1.5 (0.8–1.9) pg/mL, irisin 15.3 (14.3–16.1) μg/mL, and myostatin 3457 (2836–4712) pg/mL (Table 1).

**Figure 2:**
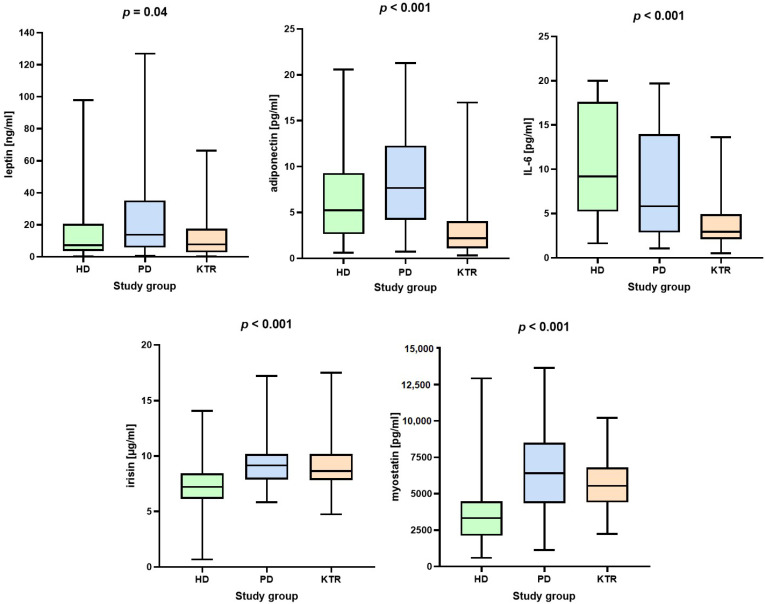
Concentrations of adipokines and myokines in the study groups.

Post-hoc analysis revealed significant differences in leptin levels between groups. In the PD patient group they were significantly higher compared to HD patients (Mean Difference (MD) = 9.9 ng/mL, *p* = 0.03) and KTR group (MD = 14.2 ng/mL, *p* = 0.005). No significant differences in leptin concentration were observed between the HD and KTR groups (MD = 4.3 ng/mL, *p* = 0.27). In the case of adiponectin, all groups differed significantly. PD patients had significantly higher levels compared to HD (MD = 2.1 pg/mL, *p* = 0.01) and KTR (MD = 4.7 pg/mL, *p* < 0.001). Adiponectin levels were significantly higher in the HD group compared to KTR (MD = 2.6 pg/mL, *p* = 0.002). IL-6 was highest in the HD group, compared to PD (MD = 2.6 pg/mL, *p* = 0.012) and KTR (MD = 6.9 pg/mL, *p* < 0.001). PD had higher IL-6 levels compared to KTR (MD = 4.3 pg/mL, *p* < 0.001). PD patients had significantly higher irisin levels compared to HD (MD = 2.2 μg/mL, *p* < 0.001), while HD had significantly lower levels compared to KTR (MD = −1.8 μg/mL, *p* < 0.001). The PD group had significantly higher myostatin levels compared to HD (MD = 2976 pg/mL, *p* < 0.001) and KTR (MD = 1216.8 pg/mL, *p* = 0.01). HD had significantly lower myostatin levels compared to KTR (MD = −1759.2 pg/mL, *p* < 0.001). The results are presented in Figure 2 and Table 3.

### 3.3. Association between Adipokines, Myokines, and Nutritional Parameters

**Figure 3:**
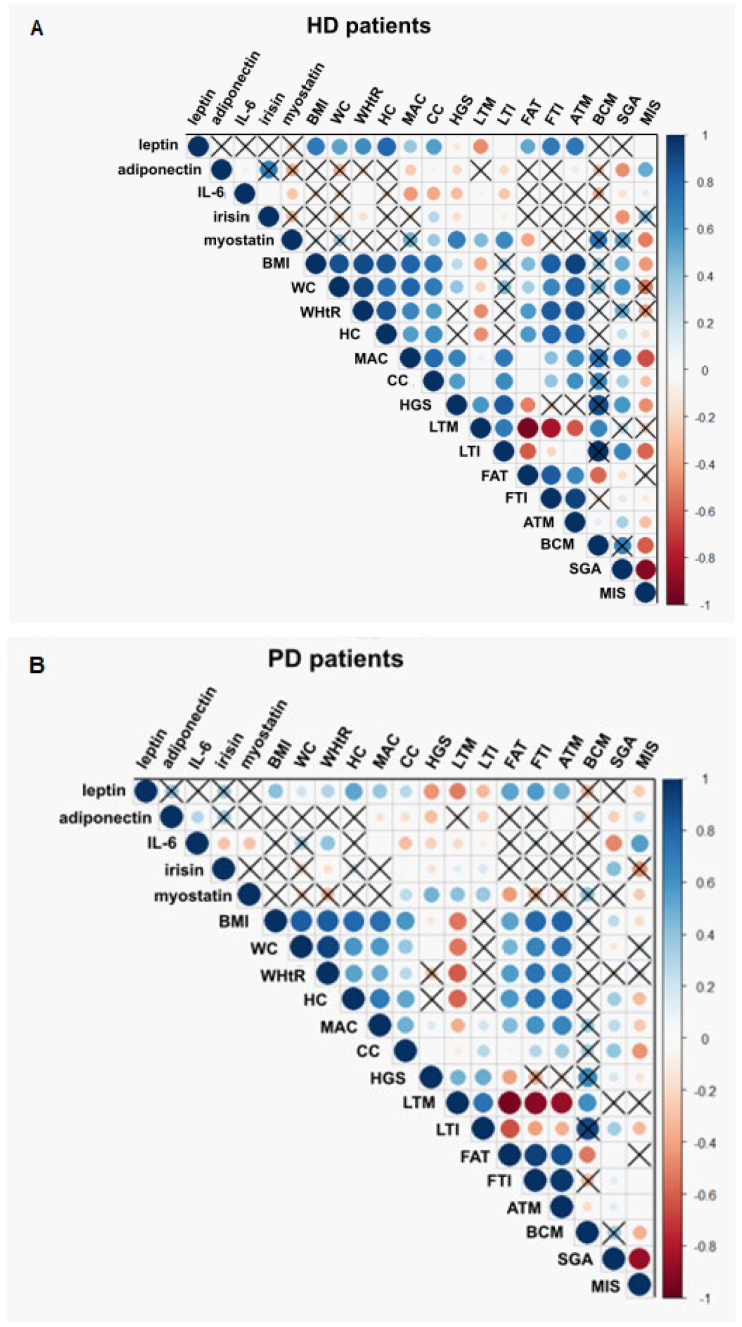

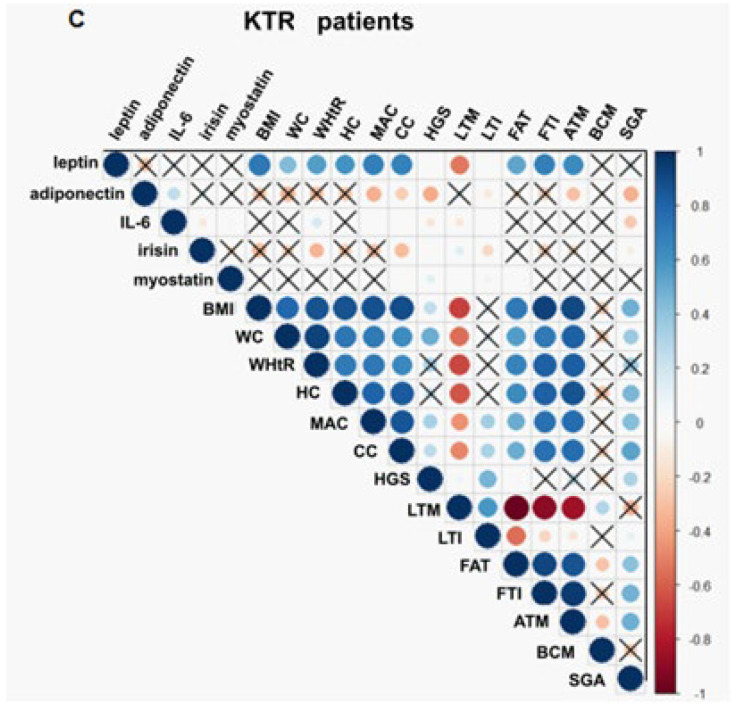
Relationship between adipokines, myokines, and nutritional status. (**A**) Relationship between adipokines, myokines, and nutritional status among HD patients. (**B**) Relationship between adipokines, myokines, and nutritional status among PD patients. (**C**) Relationship between adipokines, myokines, and nutritional status among KTR. Legend: IL-6, interleukin 6; BMI, body mass index; WC, waist circumference; WHtR, waist to height ratio; HC, hip circumference; MAC, mid-arm circumference; CC, calf circumference; HGS, hand grip strength; LTM, lean tissue mass; LTI, lean tissue index; FAT, fat mass; FTI, fat tissue index; ATM, adipose tissue mass; BCM, body cell mass; SGA, 7-Point Subjective Global Assessment; MIS, Malnutrition-Inflammation Score. Blue circles—positive correlations; red circles—negative correlations; stronger correlations are darker and larger. Black crosses represent non-significant correlations with Pearson’s statistics.

The correlations between adipokine and myokine concentration and anthropometric measurements are reported in Figure 3.

### 3.4. Adipokines and Myokines as Markers of Malnutrition and Malnutrition-Inflammation Syndrome

Overall, 32.8% of the study participants were identified as malnourished according to the 7-Point SGA. The prevalence of malnutrition was the highest in the HD group (36.9%), followed by the PD group (36.4%), while 23.1% of KT patients were classified as malnourished.

Malnourished KTRs were characterized by significantly lower leptin concentrations compared to KTRs with 7-point SGA > 5 (8.8 ± 13.4 ng/mL vs. 13.3 ± 12.7 ng/mL; *p* = 0.04). Malnourished HD patients also had lower leptin (10.6 ± 15.2 ng/mL vs. 20 ± 23 ng/mL; *p* = 0.01) and myostatin levels (3035.4 ± 1467 pg/mL vs. 4089.7 vs. 2342 pg/mL; *p* = 0.03) compared to HD patients with correct nutritional status. PD patients with SGA ≤ 5 had lower irisin concentration (8.2 ± 1.1 μg/mL vs. 10.3 ± 2.6 μg/mL; *p* = 0.002) and higher IL-6 concentration (11.1 ± 6 pg/mL vs. 6.3 ± 5.6 pg/mL; *p* = 0.006) than PD patients with normal nutritional status according to the 7-Point SGA.

**Figure 4:**
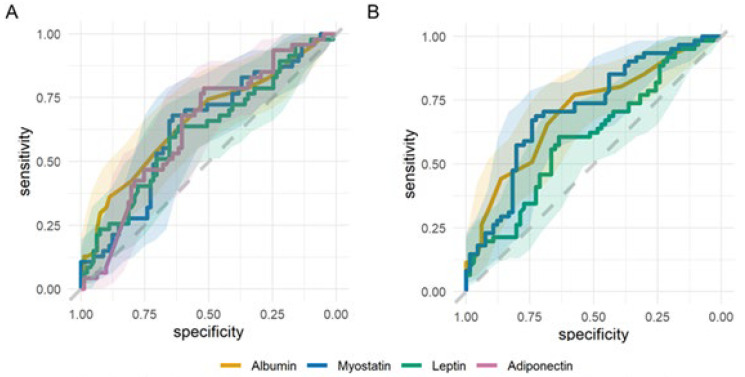
ROC curves for biomarkers of malnutrition and malnutrition-inflammation complex syndrome. (**A**) The ROC curves for albumin, myostatin, leptin, and adiponectin as biomarkers of malnutrition, assessed using the 7-Point SGA scale for all patients. Shadings represent 95% CI. (**B**) The ROC curves for albumin, myostatin, and leptin as biomarkers of MICS, assessed using MIS for dialysis patients. Shadings represent 95% CI.

The ROC analysis identified albumin, leptin, myostatin, and adiponectin as biomarkers of malnutrition diagnosed using the 7-Point SGA (Figure 4A, Table S1).

In the overall group, the highest AUC value (0.67) was obtained for albumin. In the HD and KTR groups, the highest AUC values (0.67, 0.69, respectively) were obtained for leptin. In PD patients, AUC was the highest for IL-6 and irisin (AUC 0.78, 0.75, respectively; Table S1).

Based on MIS, 47.7% of dialysis patients were diagnosed with MICS, 52.3% of those on HD, and 38.6% of the PD patients.

In the group of HD patients, it was observed that patients with MICS were characterized by higher concentrations of adiponectin (6.9 ± 4.3 μg/mL vs. 5.1 ± 4 μg/mL; *p* = 0.03) and lower concentrations of myostatin (3108.5 ± 1525 pg/mL vs. 4412.4 ± 2478.1 pg/mL; *p* = 0.006) compared to HD patients with normal nutritional status. In the group of PD patients, it was observed that patients with MICS had lower concentrations of irisin (8.5 ± 1.3 μg/mL vs. 10.2 ± 2.7 μg/mL; *p* = 0.02) and myostatin (5284 ± 2537.8 pg/mL vs. 7479.8 ± 2549.4 pg/mL; *p* = 0.01), and higher concentrations of IL-6 (12.2 ± 5.8 pg/mL vs. 5.5 ± 4.9 pg/mL; *p* < 0.001) compared to patients with MIS < 8.

Pooling dialysis patients, the biomarkers found to be significant for defining MICS (Figure 4B, Table S2) were albumin (AUC 0.7), myostatin (AUC 0.71), and leptin (AUC 0.6).

### 3.5. Adipokines and Myokines as Markers of Sarcopenia

In the whole group, 37 patients (20.6%) were diagnosed with sarcopenia on the basis of low HGS and LTI scores (28 HD, 7 PD, and 2 KTR), and 8 with sarcopenic obesity on the basis of increased BMI and low HGS and LTI scores.

In the group of HD and PD patients, those with sarcopenia were characterized by lower myostatin concentrations compared to non-sarcopenic patients (HD group: 2792.8 ± 1276 pg/mL vs. 4428.7 ± 2345 pg/mL; *p* < 0.001; PD group: 4358.7 ± 2388.8 pg/mL vs. 7436.5 ± 2475.2 pg/mL; *p* = 0.008).

**Figure 5:**
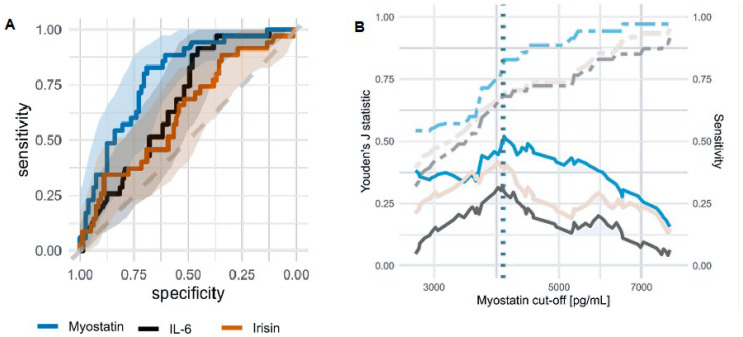
ROC curves for biomarkers of sarcopenia in dialysis patients (HD and PD) and diagnostic characteristics of myostatin. (**A**) The ROC curves for myostatin, IL-6, and irisin as biomarkers of sarcopenia in dialysis patients. Shadings represent 95% CI. (**B**) Diagnostic characteristics (Youden’s J statistic, solid line; sensitivity, dashed line) of myostatin for three conditions: sarcopenia (blue), MICS (beige), and malnutrition assessed using the 7-Point SGA (gray) among dialysis patients. Vertical lines represent cutoff values that have maximum Youden’s J statistic.

ROC analysis (Figure 5A) suggested a good diagnostic yield of myostatin (AUC 0.79), IL-6 (AUC 0.67), and irisin (AUC 0.62) among dialysis patients (KTR were not included due to the very low prevalence of sarcopenia). There were no significant differences between HD and PD patients in the AUC of the ROC curves for any of the biomarkers analyzed (Table S3). The ROC curve for serum myostatin had a significantly higher AUC than albumin (0.79 vs. 0.595) and was not superimposed on the ROC curve for albumin (Venkatraman’s test for two paired ROC curves: *p* = 0.005). Both biomarkers were characterized by high negative predictive values (>90%) and low likelihood of a negative test (Table S4).

Myostatin stands out as a significant marker for malnutrition, MICS, and sarcopenia. Figure 5B shows the cutoff points.

Table S4 shows the parameters of malnutrition and sarcopenia with the highest AUC values among dialysis patients.

### 3.6. Regression Model to Predict Sarcopenia

The initial regression analysis, including all variables, is shown in Table 4. This model shows an independent association of myostatin with sarcopenia.

The backward deletion of non-significant variables allowed us to build a final model (Table 4), which includes age, albumin, adiponectin, and myostatin concentrations as variables of interest in predicting sarcopenia. The formula resulting from the final model is as follows:

Log of odds ratio = 2.565 + Age × 0.0019 − Albumin × 0.0725 − Adiponectin × 0.0304 − Myostatin × 0.0005

where age is in years, albumin in g/dL, adiponectin in µg/mL, and myostatin in pg/mL. This formula has an AUC of 0.806 (95% CI: 0.721–0.891) with a Youden’s J index cutoff of 0.2307, with 61.54 (42.84–80.24)% positive predictive value and 79.78 (71.43–88.12)% negative predictive value.

## 4. Discussion

The main results obtained in this study, whose aim was to explore whether adipokine and myokine signatures could be used as reliable markers of malnutrition and sarcopenia, thus making a rapid diagnosis possible, in particular in settings in which there is a lack of availability of dedicated dietician time, is to propose a simple formula that predicts the likelihood of sarcopenia diagnosis in dialysis patients, whether on HD or PD.

The formula, which has an AUC of 0.806 (95% CI: 0.721–0.891), a Youden’s J index cutoff of 0.2307, a positive predictive value of 61.54%, and a negative predictive value of 79.78%, takes four factors into account: age (in years), albumin concentration (g/dL), adiponectin (µg/mL), and myostatin (pg/mL) levels.

The formula (log of odds ratio = 2.565 + Age × 0.0019 − Albumin × 0.0725 − Adiponectin × 0.0304 − Myostatin × 0.0005) was defined via backward deletion analysis of a larger set of variables, including sex, leptin, irisin, and IL-6 (Table 4). The best self-standing biomarkers of sarcopenia in dialysis patients were myostatin (AUC 0.789) and IL-6 (AUC 0.67); however, the latter was not retained in the final model.

The direction of the effect shows that higher age increases the likelihood of sarcopenia, while higher levels of albumin, adiponectin, and myostatin reduce its likelihood. Due to the very low prevalence of sarcopenia in our KTRs, these patients were not included in the model.

Conversely, no combination of biomarkers performed better than the single ones in establishing a diagnosis of malnutrition, according to SGA or MIS, and albumin, with an AUC of 0.7, and myostatin (AUC 0.71) were the single best biochemical markers of this condition, a not unexpected finding, as is the finding for albumin levels.

While this formula may not be practical for daily clinical use, it holds significant relevance in research, clinical trials, population health management, clinical decision support, and professional education. It could contribute to a better understanding of sarcopenia and potentially lead to more effective interventions and management strategies for dialysis patients.

In our population, we observed several correlations between various markers of nutritional status and the concentrations of adipokines and myokines (Figure 2). Myostatin performed well in our population, for predicting both sarcopenia and malnutrition (7-Point SGA and MIS). However, in the latter case, there was no advantage in our cohort when the diagnostic yield was compared to the one for albumin, a solid, classic, inexpensive, but yet imperfect marker of malnutrition (and of mortality) in dialysis patients [51,52].

Previous research results regarding myostatin concentration in sarcopenia have been inconclusive. In the study conducted by Yasar et al., it was found that early identification of sarcopenia benefited from assessing serum myostatin levels. However, in this study, myostatin levels were significantly higher in the sarcopenia group, while in our study myostatin concentration was inversely associated with sarcopenia [53]. Conversely, in elderly CKD patients on chronic HD, Asikin et al. found that the severity of sarcopenia was proportional to serum myostatin levels [54]. Furthermore, in keeping with our findings, Alexopoulos et al. found that low myostatin levels were independently linked to reduced skeletal muscle index and sarcopenia in patients with liver cirrhosis, another condition in which muscle mass is significantly reduced [55]. In advanced sarcopenia, where there is a significant loss of muscle mass, there may be a decrease in myostatin levels due to diminished production by the low muscle tissue [29]. In the setting of uremia toxicity, the expression of myostatin has been described as increased, and it is therefore possible that patients with better kidney function or those who have been effectively treated have lower myostatin concentrations [56].

Adiponectin was the second “nonconventional” marker retained in our formula. Adiponectin levels in patients with CKD are higher due to reduced excretion. No significant differences in adiponectin levels were observed between the groups with and without sarcopenia in our study, but this is an important element of the model contributing to the increase in AUC. Hyun et al. observed that increased adiponectin levels were independently associated with PEW [57]. The study conducted by Beberashvili et al. observed that HD patients with sarcopenic obesity had lower adiponectin concentrations compared to non-obese sarcopenic HD patients [58]. Table S5 provides a literature review regarding adipokines and myokines and nutritional status.

Regarding the ability of dialysis to remove the molecules tested in this study, leptin is cleared by hi-flux hemodialysis membranes, and hemodiafiltration seems to reduce its circulating levels [59,60]. It is not known whether PD clears leptin. Instead, hemo- or peritoneal dialysis does not seem to clear adiponectin [61].

A recent study during the COVID-19 pandemic has shown a reduction in IL-6 plasmatic values in patients dialyzed with medium cut-off membranes [62]. However, other studies have shown an increased production of IL-6 in both HD or PD patients; thus, the complex interplay between dialysis-induced inflammation and inflammatory molecule removal by dialysis is difficult to disentangle [63].

Myostatin pre-dialysis levels are higher than post-dialysis ones in hemodialyzed patients, irrespective of the hemodialysis technique [64,65]. No study specifically assessed myostatin dialyzability in PD patients. Although no direct proof exists that myostatin is dialyzed, considering its molecular weight of 25 kDa it could behave as a middle molecule, and this would explain why its levels are reduced, while remaining higher than controls, after one hemodialysis session, particularly in patients on hemodiafiltration. Irisin has a molecular weight of between 13 and 20 kDa, and no study has assessed whether it is dialyzed or not, either in hemo- or peritoneal dialysis. Once again, it could behave as a middle molecule, with its dialysis clearance depending on the dialysis technique [66]. The study of the dialyzability of this molecule is complex as some of them may be modulated during a dialysis session as the result of the activation of inflammatory pathways due to the contact of blood with the dialysis lines and dialyzer membrane. In all cases, a contribution to dialysis clearance by the absorbance properties of the dialyzer cannot be ruled out.

Our study, which has the strength of proposing a novel tool for the biochemical diagnosis of sarcopenia, yet to be validated in larger dialysis populations, has several limitations. The first, rather obvious one, is the low number of study participants, and the fact that they were recruited in a single center. However, this weakness may be at least partly counterbalanced by the fact that the biochemical analysis was centralized, patients received homogeneous care, with well-established dialysis schedules, and above all, nutritional evaluations were performed by the same skilled dietitian, thus providing uniform diagnostic definitions.

A further limitation of our study was the use of a body composition analyzer, which did not allow for the assessment of appendicular skeletal muscle mass, presently preferred as a reference for muscle mass evaluation. However, the multiple, homogeneous evaluations may at least partly compensate for this relevant bias.

Finally, other limitations include the absence of a validation group of patients to assess the external validity of the prediction model, the possibility of unmeasured confounders, and a possible selection bias because of the high refusal rate in the kidney transplant group.

While further studies on different populations are clearly needed, we considered that, in a moment when meeting the need for detailed evaluation has become difficult due to a lack of time and skilled renal dieticians, our findings could help future researchers focus on specific biomarkers and their combinations. In particular, the formula we propose could be the subject for future validation studies and might help clinicians prompt further dietary interventions in collaboration with dieticians, perhaps in tertiary centers if not available in the setting where the patient dialyzes. This could overcome the shortage of renal dieticians and benefit our dialyzed patients as well.

## 5. Conclusions

While individual laboratory markers of nutritional status cannot replace assessment by a qualified dietician, they can aid in the early detection or monitoring of nutritional deficiencies, complementing a comprehensive evaluation provided by an expert in the field. The findings and the formula described in this study, based on a combination of age, albumin, myostatin, and adiponectin levels, could be a starting point for further research on the rapid initial assessment of sarcopenia in dialysis patients.

## Figures and Tables

**Table 2:** Main characteristics of the study groups.

Baseline Data					
	All	HD	PD	KTR	*p*-Values
N	180	84	44	52	
Females/Males (*n*) (%)	80/100 (44.4%/55.6)	36/48 (42.9/57.1)	21/23 (47.7/52.3)	23/29 (44.2/55.8)	0.860
Age (in years)	56.1 ± 16.3	61.7 ± 16.4	52.2 ± 17.8	50.4 ± 11.6	0.002
Dialysis vintage (in months)	19 (9–48)	28.5 (9–72)	14 (7–29)	18 (10–36) before KT	0.040
Kt/V	-	1.7 (1.5–1.9) per session	2.3 (1.9–3.2) weekly	-	-
Ultrafiltration (mL)	-	2200 (1200–3000) per session	1000 (550–1220) per day	-	-
Anthropometric and physical data					
	All	HD	PD	KTR	*p*-values
BMI (kg/m^2^)	25.9 (22.6–28.7)	24.6 (22.4–27.7)	27.2 (23.6–29.4)	26.2 (22.8–29.6)	0.220
BMI ≥ 30 (%)	17.8	14.3	18.2	23.1	0.430
BMI < 23 (%)	29.4	35.2	20.5	28.8	0.250
MAC (cm)	28 (25–30)	26 (23–29)	28 (27–31)	29 (27–31)	<0.001
MAC < 22 cm (%)	5.6	8.3	4.5	1.9	0.260
CC (cm)	35 (32–38)	33 (30–35)	38 (36–41)	36 (33–38.8)	<0.001
CC < 31 cm (%)	18.3	32.1	4.5	7.7	<0.001
HGS (kg)	27.4± 10.8	23.2 ± 10.5	28.6 ± 10.9	33.1 ± 8	<0.001
Body composition assessed with BIA					
	All	HD	PD	KTR	*p*-values
OH (L)	0.9 (−0.2–1.9)	0.7 (−0.8–1.9)	1.35 (0.4–3.6)	0.8 (−0.1–1.7)	0.009
TBW (L)	33.3 (29–39)	32 (27.9–35.3)	37.1 (31.1–41.8)	34.1 (30.9–40.5)	0.006
ECW (L)	15.7 (13.9–19.3)	14.9 (13.2–17.1)	18.3 (14.4–20.3)	15.8 (14.6–19.1)	0.005
ICW (L)	17.7 (14.9–20.3)	16.6 (14.2–19.2)	17.9 (15.9–21)	18.6 (15.8–20.4)	0.010
LTM (%)	48.3± 12.6	48 ± 13.5	50.3 ± 12.5	47 ± 11.2	0.430
LTI (kg/m^2^)	12.1 ± 2.5	11.6 ± 2.6	13.1 ± 2.4	11.9 ± 2	0.007
FAT (%)	36.4 ± 9.9	36.6 ± 10.8	34.1 ± 9.8	38 ± 8.4	0.180
FTI (kg/m^2^)	12.6 (9.6–16.8)	12.1 (9.5–15.3)	12.8 (9.6–15.5)	12.9 (9.8–17.5)	0.500
ATM (kg)	37.8± 15.5	36.3 ± 16	36.5 ± 15	41 ± 15.2	0.160
BCM (kg)	18 (14.4–22.8)	15.9 (12.9–21.6)	19.2 (15.8–23.9)	18.8 (15–22.7)	0.030
7-Point SGA, *n* (%)					
	All	HD	PD	KTR	*p*-values
7	36 (20)	7 (8.3)	13 (29.5)	16 (30.8)	0.003
6	85 (47.2)	46 (54.8)	15 (34.1)	24 (46.2)	
5	38 (21.1)	21 (25)	10 (22.7)	7 (13.5)	
4	15 (8.3)	9 (10.7)	2 (4.5)	4 (7.7)	
3	3 (1.7)	0 (0)	3 (6.8)	0 (0)	
2	2 (1.1)	1 (1.2)	0 (0)	1 (1.9)	
1	1 (0.6)	0 (0)	1 (2.3)	0 (0)	
Well-nourished (%)	67.2	63.1	63.6457	76.9	0.210
Malnourished (%)	32.8	36.9	36.4	23.1	
Cause of CKD, *n* (%)					
	All	HD	PD	KTR	*p*-value
Glomerulonephritis	50 (27.8)	19 (22.6)	13 (29.5)	18 (34.6)	0.720
Diabetic nephropathy	32 (17.8)	15 (17.9)	10 (22.7)	7 (13.5)	
Hypertensive nephropathy	14 (7.8)	4 (4.8)	8 (18.2)	2 (3.8)	
ADPKD	23 (12.8)	12 (14.3)	3 (6.8)	8 (15.4)	
Other	54 (30)	31 (36.9)	7 (15.9)	16 (30.8)	

Legend: Kt/V, dialysis adequacy; BMI, body mass index; MAC, mid-arm circumference; CC, calf circumference; HGS, hand grip strength; BIA, bioelectrical impedance analysis; OH, overhydration; TBW, total body water; ECW, extracellular water; ICW, intracellular water; LTM, lean tissue mass; LTI, lean tissue index; FAT, fat mass; FTI, fat tissue index; ATM, adipose tissue mass; BCM, body cell mass; 7-Point SGA, 7-Point Subjective Global Assessment; CKD, chronic kidney disease; ADPKD, Autosomal Dominant Polycystic Kidney Disease.

**Table 3:** Main biochemical data for the study population.

Basic Biochemical Data						
Parameters	References Value	All	HD Patients	PD Patients	KTR	*p*-Values
N		180	84	44	52	
Creatinine (mg/dL)	0.7–1.2	5.3 (2.2–8.4)	7.2 (5.0–9.1)	7.7 (5.2–10.1)	1.3 (1.04–1.9)	<0.001
eGFR CKD-EPI (mL/min/1.73 m^2^)	>90	-	-	-	53.5 (39–69.5)	-
BUN (mg/dL)	8.4–25.7	47.7± 16.3	52.3 ± 14.3	49.4 ± 14.2	31.4 ± 15	<0.001
Calcium (mg/dL)	8.9–10	9 (8.4–9.5)	8.8 (8.3–9.3)	8.8 (8.4–9.2)	9.7 (9.4–10)	<0.001
Phosphorus (mg/dL)	2.3–4.7	5 (3.7–6.3)	5.2 (3.9–6.5)	5.8 (4.8–6.9)	2.9 (2.5–3.3)	<0.001
Sodium (mmol/L)	135–145	139 (137–141)	138 (135–141)	140 (138–141.5)	140 (139–141.5)	0.004
Potassium (mmol/L)	3.5–5.1	4.8 (4.3–5.4)	5.3 (4.7–5.7)	4.5 (4.1–5.3)	4.3 (4.1–4.8)	<0.001
Hemoglobin (g/dL)	12–15 for F, 13–17 for M	11.1 (10.1–13.1)	10.3 (9.7–11.1)	11.1 (10.3–12.2)	14.1 (12.7–15)	<0.001
Biochemical markers of nutritional status						
	References value	All	HD	PD	KTR	*p*-values
Albumin (g/dL)	3.8–5.2	3.5 (3.2–4)	3.4 (3.1–3.6)	3.35 (2.95–3.7)	4.1 (4–4.35)	<0.001
Albumin level <3.8 (%)		63.9	86.9	86.4	7.7	<0.001
Albumin level <3.5 (%)		42.8	61.9	52.3	3.8	<0.001
Transferrin (mg/dL)	200–400	173 (155–199)	166 (149–189)	195 (170–221)	-	<0.001
Total cholesterol (mg/dL)	115–190	176 (148–219)	160 (127–196)	209.5 (172.5–256.5)	186.5 (162–207)	<0.001
HDL cholesterol (mg/dL)	>45 for F, >40 for M	44 (36–56)	40 (35–51)	41.5 (36–53)	52 (44–59.5)	<0.001
Total number of lymphocytes (/1 mm^3^)	1–3	1.5 (1.1–2.1)	1.3 (1–1.7)	1.3 (1.1–1.7)	2.3 (1.8–2.8)	<0.001
Adipokines and myokines						
		All	HD patients	PD patients	KTR	*p*-values
Leptin (ng/mL)		10.1 (3.7–22.6)	7.2 (3.6–19.9)	13.8 (6.2–34.4)	7.8 (2.8–17.1)	0.040
Adiponectin (μg/mL)		4.3 (2–9.4)	5.3 (2.7–9.3)	7.7 (4.3–12.3)	2.2 (1.1–4)	<0.001
IL-6 (pg/mL)		5.9 (2.8–13.3)	9.2 (5.3–17.6)	5.8 (2.9–13.9)	2.9 (2.1–4.9)	<0.001
Irisin (μg/mL)		8.1 (7.1–9.5)	7.2 (6.2–8.4)	9.1 (7.9–10.2)	8.6 (7.8–10.2)	<0.001
Myostatin (pg/mL)		4448 (3047.4–6438)	3334 (2149–4460)	6418 (4366–8396)	5536 (4406–6730)	<0.001

eGFR CKD-EPI, estimated glomerular filtration rate calculated using the Chronic Kidney Disease Epidemiology Collaboration equation; BUN, blood urea nitrogen; HDL, high-density lipoprotein; IL-6, interleukin 6.

**Table 4:** Multivariable stepwise backward deletion logistic regression analysis estimating the effects of various predictors on sarcopenia.

		95% CI		
	Estimates	Lower	Higher	*p*-Values
**Initial model**				
Age (in years)	0.019	−0.012	0.051	0.231
Sex (men)	−0.137	−1.311	1.037	0.819
Albumin (g/dL)	−0.065	−0.176	0.045	0.247
Leptin (ng/mL)	0.005	−0.017	0.026	0.667
Adiponectin (µg/mL)	−0.022	−0.133	0.089	0.155
Irisin (μg/mL)	−0.072	−0.296	0.151	0.526
Myostatin (pg/mL)	−0.005	−0.001	0.001	0.002
IL-6 (pg/mL)	0.004	−0.077	0.085	0.917
**Final model**				
Age (in years)	0.0019	−0.009	0.048	0.19
Albumin (g/dL)	−0.0725	−0.177	0.032	0.17
Adiponectin (µg/mL)	−0.0304	−0.137	0.0758	0.57
Myostatin (pg/mL)	−0.0005	−0.0008	−0.0002	0.0004

## Data Availability

The data sets used and/or analyzed during the current study are available from the corresponding author upon reasonable request.

## Supplementary Materials

The following supporting information can be downloaded at: https://www.mdpi.com/article/10.3390/nu16152480/s1, References [67,68,69,70,71,72,73,74,75,76,77,78,79] are cited in SM file. Table S1: Biomarkers of malnutrition assessed via SGA; Table S2: Biomarkers of MICS assessed using MIS; Table S3: Biomarkers of sarcopenia; Table S4: Biomarkers of malnutrition and sarcopenia among dialysis patients; Table S5: Review of the literature on the relationship between adipokines/myokines and nutritional status.
