# Supplementary material for: Adipokines and Myokines as Markers of Malnutrition and Sarcopenia in Patients Receiving Kidney Replacement Therapy: An Observational, Cross-Sectional Study
Source: Nutrients. 2024 Jul 31;16(15):2480. doi: 10.3390/nu16152480 (PMC11314363; doi:10.3390/nu16152480)
Supplement: Supplementary file 1 [file nutrients-16-02480-s001.zip › nutrients-3099329-supplementary.pdf]

Table S1. Biomarkers of malnutrition assessed via SGA.

Biomarker	Group of Patients					Differences between Groups	
	CKD (All)	Dialysis			KTR	PD-HD *p*-Value	Dialysis-KTR *p*-Value
		All	HD	PD			
**Albumin (↓)**							
AUC (95%)	0.6657 (0.579–0.7525)	0.6642 (0.564–0.765)	0.648 (0.523–0.773)	0.6964 (0.523–0.870)	0.5979 (0.403–0.793)	0.66	0.56
Cutoff (sp, sen)	34.5 (65.83, 61.02)	33.5 (61.25, 63.83)	34.5 (46.15, 77.42)	27.5 (92.86, 43.75)			
Leptin (↓)							
AUC (95%)	0.622 (0.531–0.713)	0.6179 (0.516–0.720)	0.6652 (0.544–0.787)	0.531 (0.346–0.716)	0.6896 (0.477–0.902)	0.24	0.55
Cutoff (sp, sen)	9.76 (59.50, 66.10)	9.76 (61.73, 63.83)	8.015 (60.38, 74.19)	-	3.86 (77.5, 66.67)		
Adiponectin (↑)							
AUC (95%)	0.636 (0.549–0.723)	0.637 (0.539–0.735)	0.6223 (0.5–0.745)	0.6641 (0.495–0.833)	0.5104 (0.309–0.712)	0.70	0.27
Cutoff (sp, sen)	4.41 (61.16, 66.10)	4.41 (51.85, 78.72)	4.32 (56.60, 74.19)	5.73 (53.57, 87.5)			
Irisin (↓)							
AUC (95%)	-	-	0.3956 (0.268–0.524)	0.779 (0.641–0.918)	0.6208 (0.442–0.800)	<0.001	-
Cutoff (sp, sen)	-	-	-		9.28 (67.5, 58.33)		
Myostatin (↓)							
AUC (95%)	0.64 (0.553–0.727)	0.6302 (0.530–0.731)	0.6391 (0.518–0.760)	0.6362 (0.458–0.814)	0.5833 (0.392–0.775)	0.98	0.67
Cutoff (sp, sen)	3964.8 (71.07, 59.32)	3964.8 (64.20, 68.09)	3974.8 (50.94, 87.10)	5872 (67.8, 62.5)			
IL-6 (↑)							
AUC (95%)	-	-	0.539 (0.4054–0.6725)	0.7545 (0.611–0.898)	0.656 (0.463–0.850)	0.033	-
Cutoff (sp, sen)	-	-	-	4.405 (53.5, 93.75)	5.485 (87.5; 50.0)		

Legend:AUC, area under the curve; CKD, chronic kidney disease; HD, hemodialysis; PD, peritoneal dialysis; KTR, kidney transplant recipients, IL-6—interleukin 6, ↑, higher concentration; ↑, lower concentration

Table S2. Biomarkers of MICS assessed using MIS.

Biomarker	Group of Patients			Differences between Groups
	Dialysis (All)	HD	PD	*p*-Value
		Albumin (↓)		
AUC (95%)	0.7073 (0.6168–0.7978)	0.7031 (0.5899–0.8162)	0.744 (0.5849–0.9031)	0.68
Cutoff (sp, sen)	34.5 (57.58, 77.05)	34.5 (56.41, 79.55)	31.5 (85.19, 64.71)	
		Leptin (↓)		
AUC (95%)	0.6043 (0.5056–0.7031)	0.6238 (0.5024–0.7452)	0.5534 (0.3761–0.7306)	0.52
Cutoff (sp, sen)	9.965 (63.64, 60.66)	8.015 (61.54, 63.64)	-	
		Adiponectin (↑)		
AUC (95%)	0.5946 (0.4955–0.6937)	0.6369 (0.5149–0.759)	0.5708 (0.3948–0.7468)	0.55
Cutoff (sp, sen)	-	4.32 (61.54, 68.18)	-	
		Irisin (↓)		
AUC (95%)	-	0.4604 (0.3341–0.5867)	0.7059 (0.5489–0.8629)	0.019
Cutoff (sp, sen)	-	-	9.74 (51.85, 88.24)	
		Myostatin (↓)		
AUC (95%)	0.7125 (0.6222–0.8027)	0.6754 (0.5578–0.793)	0.7211 (0.557–0.8852)	0.66
Cutoff (sp, sen)	3964.8 (72.73, 68.85)	3896.8 (58.97, 77.27)	6940 (62.96, 76.47)	
		IL-6 (↑)		
AUC (95%)	-	0.6052 (0.4825–0.7279)	0.8322 (0.7079–0.9566)	0.012
Cutoff (sp, sen)	-	6.925 (48.72, 72.73)	7.48 (81.48, 76.47)	

Legend:AUC, area under the curve; HD, hemodialysis; PD, peritoneal dialysis, IL-6—interleukin 6, ↑, higher concentration; ↑, lower concentration

Table S3. Biomarkers of sarcopenia.

Biomarker	Group of Patients			Differences between HD and PD Group
	Dialysis (All)	HD	PD	*p*-Value
		Albumin (↓)		
AUC (95% CI)	0.595 (0.482–0.708)	0.597 (0.464–0.730)	0.621 (0.357–0.886)	0.87
Cutoff (sp, sen)	-	-	27.5 (84.85, 42.86)	
		Leptin (↑)		
AUC (95% CI)	0.534 (0.417–0.652)	0.537 (0.399–0.674)	0.649 (0.408–0.891)	0.43
Cutoff (sp, sen)	-	-	13.12 (57.58, 85.71)	
		Adiponectin (↓)		
AUC (95% CI)	0.533 (0.416–0.650)	0.487 (0.349–0.625)	0.522 (0.224–0.819)	0.84
		Irisin (↓)		
AUC (95% CI)	0.623 (0.512–0.734)	0.560 (0.423–0.696)	0.558 (0.246–0.871)	0.99
Cutoff (sp, sen)	6.12 (88.89, 34.29)	-	-	
		Myostatin (↓)		
AUC (95% CI)	0.789 (0.703–0.875)	0.7366 (0.6231–0.850)	0.827 (0.628–1)	0.44
Cutoff (sp, sen)	3993 (69.14, 82.86)	3993 (54.17, 89.29)	5274 (81.82, 85.71)	
		IL-6 (↑)		
AUC (95% CI)	0.670 (0.571–0.769)	0.615 (0.488–0.742)	0.725 (0.537–0.913)	0.34
Cutoff (sp, sen)	5.31 (45.68, 91.43)	5.73 (41.67, 89.29)	4.41 (48.48, 1.00)	

Legend: AUC, area under the curve; HD, hemodialysis; PD, peritoneal dialysis, IL-6—interleukin 6, ↑, higher concentration; ↑, lower concentration

Table S4. Biomarkers of malnutrition and sarcopenia among dialysis patients.

**Characteristic**	**Clinical definition**				
	**Malnutrition (7-Point SGA)**	**Malnutrition-Inflammation Complex Syndrome (MIS)**		**Sarcopenia**	
	**Albumin (<33.5 g/L)**	**Albumin (<34.5 g/L)**	**Myostatin (<3964.8 pg/mL)**	**IL-6 (>5.31 pg/mL)**	**Myostatin (<3993 pg/mL)**
Number of patients, n (%)	61 (48.0)	75 (59.1)	60 (47.2)	76 (65.5)	54 (46.6)
Sensitivity, %	63.8 (48.5–77.3)	77.0 (64.5–86.8)	68.9 (55.7–80.1)	91.4 (76.9–98.2)	82.9 (66.4–93.4)
Specificity, %	61.3 (49.7–71.9)	57.6 (44.8–69.7)	72.7 (60.4–83.0)	45.7 (34.6–57.1)	69.1 (57.9–78.9)
Diagnostic accuracy, %	62.2 (53.2–70.7)	66.9 (58.0–75.0)	70.9 (62.1–78.6)	59.5 (50.0–68.5)	73.3 (64.3–81.1)
Positive predictive value, %	49.2 (36.1–62.3)	62.7 (50.7–73.6)	70.0 (56.8–81.2)	42.1 (30.9–54.0)	53.7 (39.6–67.4)
Negative predictive value, %	74.2 (62.0–84.2)	73.1 (59.0–84.4)	71.6 (59.3–82.0)	92.5 (79.6–98.4)	90.3 (80.1–96.4)
Proportion of false positives, %	38.8 (28.1–50.3)	42.4 (30.3–55.2)	27.3 (17.0–39.6)	54.3 (42.9–65.4)	30.9 (21.1–42.1)
Proportion of false negative, %	36.2 (22.7–51.5)	23.0 (13.2–35.5)	31.1 (19.9–44.3)	8.6 (1.8–23.1)	17.1 (6.6–33.6)
False Discovery Rate, %	50.8 (37.7–63.9)	37.3 (26.4–49.3)	30.0 (18.8–43.2)	57.9 (46.0–69.1)	46.3 (32.6–60.4)
False Omission Rate. %	25.8 (15.8–38.0)	26.9 (15.6–41.0)	28.4 (18.0–40.7)	7.5 (1.6–20.4)	9.7 % (3.6–19.9)
Youden's J statistic	0.251 (−0.0178–0.493)	0.346 (0.0929–0.565)	0.416 (0.161–0.631)	0.371 (0.115–0.553)	0.520 (0.242–0.724)
Likelihood ratio of a positive test	1.647 (1.1612–2.337)	1.816 (1.3285–2.483)	2.525 (1.645–3.876)	1.683 (1.345–2.106)	2.685 (1.875–3.844)
Likelihood ratio of a negative test	0.591 (0.3888–0.897)	0.399 (0.2407–0.660)	0.428 (0.287–0.640)	0.188 (0.062–0.568)	0.248 (0.118–0.521)

Legend:SGA, subjective global assessment, IL-6—interleukin 6.

Table S5. Review of the literature on the relationship between adipokines/myokines and nutritional status.

Study	Study Population	Characteristics of Study Population/Treatment	Sample Size	Adipokine/ Myokine	Measurement Method	Concentration	Results
Kaynar K, 2014 [67]	HD	Age 9.8 ± 16.1 years	30	Leptin (L) Adiponectin(A)	Serum, ELISA kit (DRG, Marburg, Germany and eBioscinence San Diego, CA, USA)	L 20.8 ± 31.7 ng/mL; A 49,665.7 ± 35,010.7 ng/mL L 27.0 ± 31.6 ng/mL; A 34,626.5 ± 33,635.0 ng/mL L 27.0 ± 31.6 ng/mL; A 34,626.5 ± 33,635.0 ng/mL L 11.5 ± 13.6 ng/mL; A 15,242.3 ± 10,306.7 ng/mL L 13.6 ± 11.9 ng/mL; A 17,627.6 ± 11421.1 ng/mL	Serum leptin levels were positively correlated with total skinfold thickness; the presence of PEW was significantly negatively correlated with serum leptin levels and positively correlated with serum adiponectin levels in PD patients; adiponectin levels were positively correlated with the presence of PEW (in the whole population)
	PD	Age 39.1 ± 13.4 years	30				
	Pre-dialysis group	CKD stage 4 or 5, age 58.4 ± 15.2 years	30				
	KTR	Age 47.1 ± 13.1 years	30				
	Control group	Healthy volunteers, age 33.4 ± 9.4 years	30				
Małgorzewicz S, 2014 [68]	KTR	Age 52.4 ± 14.0 years	80	Leptin Adiponectin	Serum ELISA kit (DRG, Germany, and Linco Research, St Charles, Missouri, USA)	20.4 ± 24.3 µg/mL 10.1 ± 5.2 mg/L	Leptin concentration correlated positively with BMI and percentage of fat mass and negatively with nutritional status assessed using SGA
Feret W, 2022 [69]	HD	Age median: 65; IQR = 21 years	47	Leptin IL-6	Leptin-serum ELISA kit (Euroimmun, Poland) IL-6-Luminex kits (Biotechne, Poland)	16.4 (IQR 51.8) ng/mL 3 (IQR 2.9) pg/mL	Leptin level was associated with percentage of muscle mass, visceral adipose tissue, and BMI; IL-6 was negatively correlated with phase angle
Machiba Y, 2018 [70]	HD	Age 61.6 ± 10.8 years	113	Adiponectin	Serum ELISA kit (Otsuka Pharmaceuticals Co., Tokyo, Japan)	17.6 (11.7–24.5) µg/mL	Serum adiponectin was negatively correlated with total fat mass and percentage of truncal fat
Lee MJ, 2015 [71]	PD	Age 54.1 ± 11.6 years	102	Irisin	Serum, ELISA kit (Adipogen, San Diego, CA, USA)	184.2 ± 88.0 ng/mL 457.2 ± 105.5 ng/mL	Serum irisin was positively correlated with mid-arm muscle circumference and thigh circumference
	Control	Healthy volunteers, age 54.1 ± 11.5 years	35				
He WY, 2016 [72]	HD	Age 60 ± 15 years	128	Irisin	Serum, ELISA kit (Phoenix Pharmaceuticals, Burlingame, CA, USA)	76.5 ± 81 ng/mL 727.8 ± 95 ng/mL	Serum irisin levels were lower in HD patients with PEW; there was a positive correlation between irisin and muscle mass
	Control	Healthy volunteers, age 58 ± 14 years	40				
Kałużna M, 2016 [73]	HD	Age 56.5 (40.0–66.5) years	41	Irisin		Dialysis 4.6 (3.5–6.4) µg/mL	

	PD	Healthy volunteers, age 54 (48.0–59.0) years	7		Serum, ELISA kit (AdipoGen)	Control 7.9 (6.5–9.5) µg/mL	There was no significant link between irisin levels and body composition
	Control		36				
Yilmaz H, 2016 [74]	HD	Non-diabetic, age 52.5 ± 12.1 years	108	Irisin	Serum, ELISA kit (Aviscera Biosciences, Santa Clara, CA)	523.5 ± 229.3 ng/mL<br>511.3 ± 259.7 ng/mL	Irisin levels were associated with total fat mass and waist circumference
	Control	Age 52.9 ± 12.8 years	40				
Zhou S, 2024 [75]	PD	Age 58.1 ± 13.2 years	154	Irisin	Serum, ELISA kit (Phoenix Pharmaceutical, Burlingame, CA, USA)	Median 113.5 ng/mL (interquartile range, 106.2–119.8 ng/mL)	Serum irisin was positively correlated with muscle mass and negatively correlated with percentage of body fat
Demir C, 2023 [76]	HD	Age 42.0 ± 14.0 years	23	Irisin	Serum, ELISA kit (Elabscience, Houston, Texas, USA)	426.6 ± 191.2 pg/mL	İrisin was positively correlated with BMI in HD patients
	KTR	Age 45.8 ± 10.1 years	26			342.6 ± 174.8 pg/mL	
	Control	Age 39.4 ± 8.0 years	25			208.0 ± 186.1 pg/mL	
Hou YC, 2023 [77]	Control	eGFR > 60 mL/min	16	Irisin	Serum, ELISA (Abbkine, Wuhan, China)	Control<br>Myostatin<br>2.6 ± 4.5 ng/mL<br>HD<br>Irisin<br>72.7 ± 59.6 pg/mL<br>Myostatin<br>1.3 ± 2.0 ng/mL<br>IL-6 4.7 ± 4.7 pg/mL	Higher irisin levels (>63 pg/mL) were negatively correlated with low HGS; the concentrations of irisin, myostatin, and IL-6 were not predictive of sarcopenia; myostatin was higher in the control group (without statistic difference)
	CKD	eGFR 15–60 mL/min	17	Myostatin			
	HD	HD three times per week with duration 3–4 h for each session	41	IL-6			
Lee SM, 2019 [78]	HD	Age 58.9 ± 11.2 years	37	Myostatin	ELISA (R&D Systems Inc., Minneapolis, NM, USA)	5.3 ± 2.6 ng/mL	Myostatin was positively correlated with appendicular skeletal muscle index
	PD		34				
Delanaye P, 2019 [79]	HD	Age 71 (58; 81) years	204	Myostatin	Serum, ELISA kit (R&D Systems, Minneapolis, MN, USA)	2573 (1662; 3703) pg/mL	The significant associations between HGS and myostatin; the ability of myostatin to detect a decreased HGS: AUC 0.72 (95% CI: 0.65–0.79); serum myostatin was positively correlated with muscle mass index

Legend: HD, hemodialysis; PD, peritoneal dialysis; CKD, chronic kidney disease; KTR, kidney transplant recipients; ELISA, enzyme-linked immunosorbent assay; L, leptin; A, adiponectin; PEW, protein energy wasting; BMI, body mass index; SGA, Subjective Global Assessment; IL-6, interleukin 6; HGS, hand grip strength.
